# Intentional cranial modifications in the Americas: The temporal and spatial patterns of potential transmissions and cultural innovations

**DOI:** 10.1016/j.isci.2026.115643

**Published:** 2026-04-08

**Authors:** Stacey M. Ward, Marcelo R. Sánchez-Villagra, Caitlin Raymond, Gizéh Rangel-de Lazaro, Sinéad Lynch, Laura A.B. Wilson

**Affiliations:** 1 School of Archaeology and Anthropology, College of Arts and Social Sciences, The Australian National University, Canberra, ACT 2601, Australia; 2 Department of Paleontology, University of Zurich, Karl-Schmid-Strasse 4, Zürich 8006, Switzerland; 3 Art History Department, Faculty of Philosophy and Letters, University of Malaga, 29010 Malaga, Spain; 4 School of Arts, College of Humanities, SOAS, University of London, WC1H 0XG London, UK; 5 Institute of Earth Sciences, University of Lausanne, Géopolis, Lausanne 1015, Switzerland; 6 School of Biological, Earth and Environmental Sciences, UNSW Sydney, High Street, Kensington, NSW 2052, Australia; 7 ARC Training Centre for Multiscale 3D Imaging, Modelling and Manufacturing, Research School of Physics, The Australian National University, Canberra, ACT 2601, Australia

**Keywords:** Geography, Anthropology, Archeology

## Abstract

For at least 10,000 years, people from many cultures have demonstrated social status or group affiliation by performing intentional cranial modification (ICM), altering head shape for life in early infancy. We present a database of ICM cases (*n* = 1,772) across the Americas, where this practice was prominent and widespread, and use spatial statistics to explore spatiotemporal variations in the prevalence of ICM in this region from a cultural evolutionary perspective. Prediction surfaces produced using Empirical Bayesian Kriging suggested that ICM first occurred in Central America. The spatial extent of this practice then broadened to encompass North and South Americas. Spatial analyses suggest that annular ICM cases appeared where tabular cases were present and its geographic range increased rapidly relative to tabular ICM. This suggests that annular ICM may be a derived cultural behavior and that existing familiarity with cranial modification behaviors aided the adoption of additional methods of head shaping.

## Highlights

• Exploring spatiotemporal distribution of cranial modification in the Americas
• Spatial discontinuities in distribution suggest temporal variation in adoption
• Annular style appears after tabular and its spatial extent increases more rapidly
• Use of annular style aided by prior use of modification; suggests derived behavior

## Graphical abstract

**Figure:**
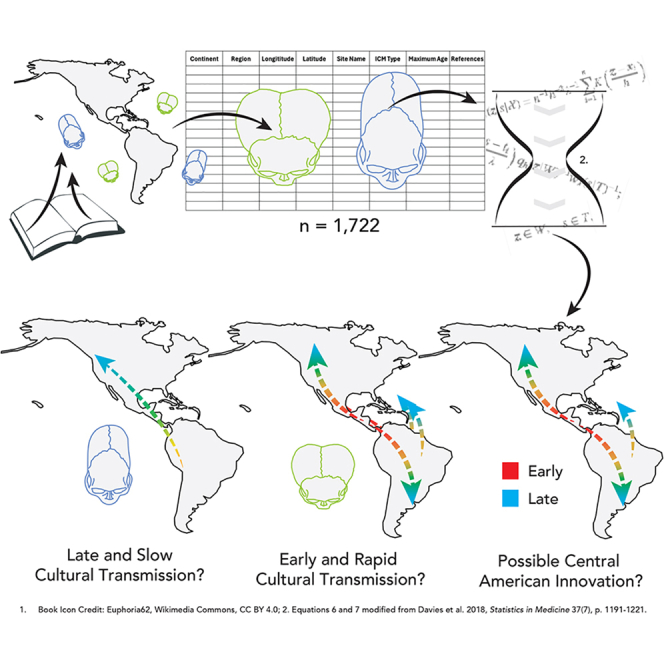

## Introduction

Intentional cranial modification (ICM) is a human cultural practice whereby the morphology of the cranium is altered in early childhood to create a permanent and highly visible marker of social identity.^1^ ICM is a deliberate, cultural practice using devices like boards or bandages to reshape the skull, in contrast to unintentional modifications that result from the use of cradleboards, byproducts of child-rearing that produce less distinct changes to the bone structure. Human remains provide an enduring record of this behavior, forming a unique, deep-time record of change over time in human cultural practices.^2^ Here, we use spatial statistics to explore diachronic change in the spatial distribution of ICM and generate hypotheses about the processes that may have shaped that distribution from a cultural evolutionary perspective.

Ethnographic and archaeological evidence of ICM, including human remains and remnants of head-shaping apparatus, suggests that the direction of cranial growth was altered by applying pressure to the cranium with long strips of cloth, boards, pads, cushions, stones, or cradles/carriers until cranial ossification was complete.^1^^,^^2^^,^^3^^,^^4^^,^^5^^,^^6^^,^^7^ As the head-shaping process requires the practitioner to be close to the child over several years, close kin likely completed cranial modification as part of traditional child-rearing practices.^1^^,^^6^ The purpose of head shaping varied according to culture and region.^8^ The practice has been used, for example, to demarcate different groups in society, as in some Andean^9^ and Maya^10^ cultures, and to signify high social status (Muisca cultures, Colombia^11^). The practice may have served as a symbol of nobility or a distinction for the ruling classes.^12^

ICM has received extensive attention over the last century, with early research focusing on the pathological impacts of ICM and the description and classification of cranial shapes.^4^^,^^13^ Key paleopathology texts^14^ have characterized cranial modification as “chronic, low-grade” trauma resulting in several sequelae, such as asymmetry and deformation of brain structures, neurological damage, and dental malocclusion.^1^^,^^15^^,^^16^^,^^17^ However, head shaping does not result in premature synostosis or reduction in the magnitude of brain growth,^14^^,^^18^ suggesting that the impacts of ICM are largely cosmetic in nature.^14^^,^^16^ Early classification schemes, which sought to link cranial morphology to head shaping methods, typically identify two overarching forms of cranial modification based on the materials and methods used for head shaping: (1) tabular or fronto-occipital modification, where boards were placed at the front and back of the skull to produce antero-posterior flattening and lateral bulging of the head, and (2) annular modification, where the head was wrapped tightly with a bandage to produce a conical cranial vault (e.g., works by Dembo and Imbelloni^4^; and Anton^19^). The outcome of modification on the developing skull is complex, with variations in the pressure, type, number, and angle of binding materials giving rise to several subtypes of cranial modification within these two major categories.^20^^,^^21^^,^^22^^,^^23^ While the use of these subtypes has fluctuated through time, the use of the “base” types of annular and tabular ICM has persisted over thousands of years, likely reflecting the simplicity of each modification mechanism (compression vs. wrapping).^3^ We focus on these core forms of ICM to provide a long-term perspective on the use of cranial modification.

The classifications of ICM formed the basis of works exploring the origins and dispersal of this practice across large regions (e.g., Dembo and Imbelloni (1938) and Lekovic et al. (2007)^4^^,^^13^). These syntheses were strongly rooted in the diffusionist perspectives of the early 20^th^ century and acknowledged neither human agency nor Indigenous autonomy and authority in shaping culture change^24^; these models have since been superseded by local and regional models based on more diverse classifications of the practice (e.g., for South America^25^ and Caribbean^26^^,^^27^). Several authors hypothesize that the practice reached the Americas either via northeast Asia^28^ or via Polynesian contact with the Americas^8^^,^^13^^,^^25^^,^^29^; others have proposed that independent centers of development were present in Eurasia and the Americas.^30^

Despite extensive research interest in ICM, there has been a lack of critical data compilation and quantitative analyses at large scale. As such, fundamental questions about processes driving variation in the spatiotemporal distribution of this cultural practice remain unanswered. We present a comprehensive database of archaeological cases of ICM (≤ 1,900 CE) in the Americas and statistical analyses exploring spatiotemporal patterns in these data. We specifically aim to (1) characterize diachronic variations in the spatial distribution of ICM in the Americas; (2) explore space-time relationships between the types of cranial modification; and (3) generate hypotheses regarding the cultural evolutionary processes underlying changing ICM distributions (cultural transmission vs. independent innovation). Our analysis of these long-term data represents a rare case study of the transmission of cultural traits in deep time and across large geographic areas based on material evidence, addressing key challenges set out for cultural evolution.^31^ Further, our statistics-driven approach provides a data-based assessment of this subject, generating broad, region-level hypotheses that future studies can individually test through further contextualization.

Our study was conducted in two major phases. First, a comprehensive literature search was undertaken and a database of ICM cases was created, and in the second phase, the constructed database was used to perform a spatiotemporal analysis of ICM.

## Results

### Spatial data exploration

**Figure 1:**
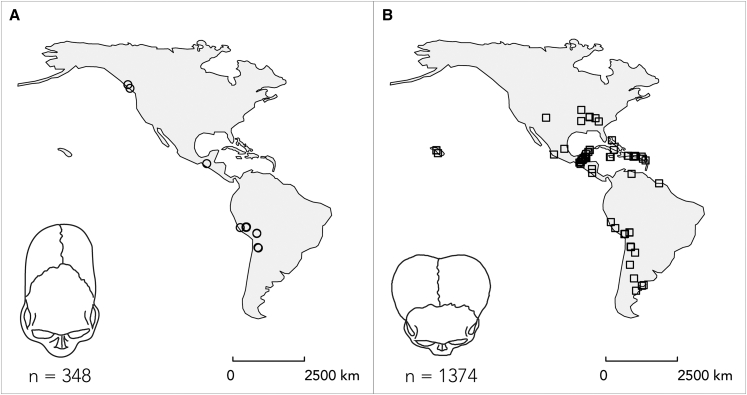
Geographic distribution of ICM cases included in this study from across the Americas (A) Geographic distribution of annular ICM cases. (B) Geographic distribution of tabular ICM cases. Scale bar represents 2,500 km.

Our database comprises a total of 1,772 individual records from the Americas (Figure 1 and Table S1). The oldest occurrence of tabular ICM in the Americas in our database dates to 2,849 BP, 2,799 years before the youngest record of tabular ICM at 50 BP and 799 years before the first occurrence of annular ICM at 2,050 BP (Figure S1 and Table S1). The youngest case of annular ICM appeared 1,900 years after the oldest case, at 150 BP. Following descriptive analysis, our database was entered into spatial analysis workflows (see Figure S2 for overview).

### Assessment of spatial autocorrelation and non-stationarity

Evaluation of the inhomogeneous *K* function demonstrated that the spatial distribution of ICM cases is both spatially autocorrelated and non-stationary (Figure S3). The inhomogeneous cross-*K* function indicates that tabular ICM influenced the presence of annular ICM and that this influence is non-stationary (Figure S4). A Moran’s *I* test on the dependent variable (maximum age of ICM occurrence) identified that ICM occurrences of a similar age tended to be correlated with one another (*p* < 0.001) (Table S2).

### Spatiotemporal kernel density estimation

The annular ICM sample size (*n* = 348) was too small and sparsely distributed across the Americas to support spatiotemporal kernel density estimation (StKDE). We, therefore, present StKDE results for only the combined (annular and tabular) sample.

**Figure 2:**
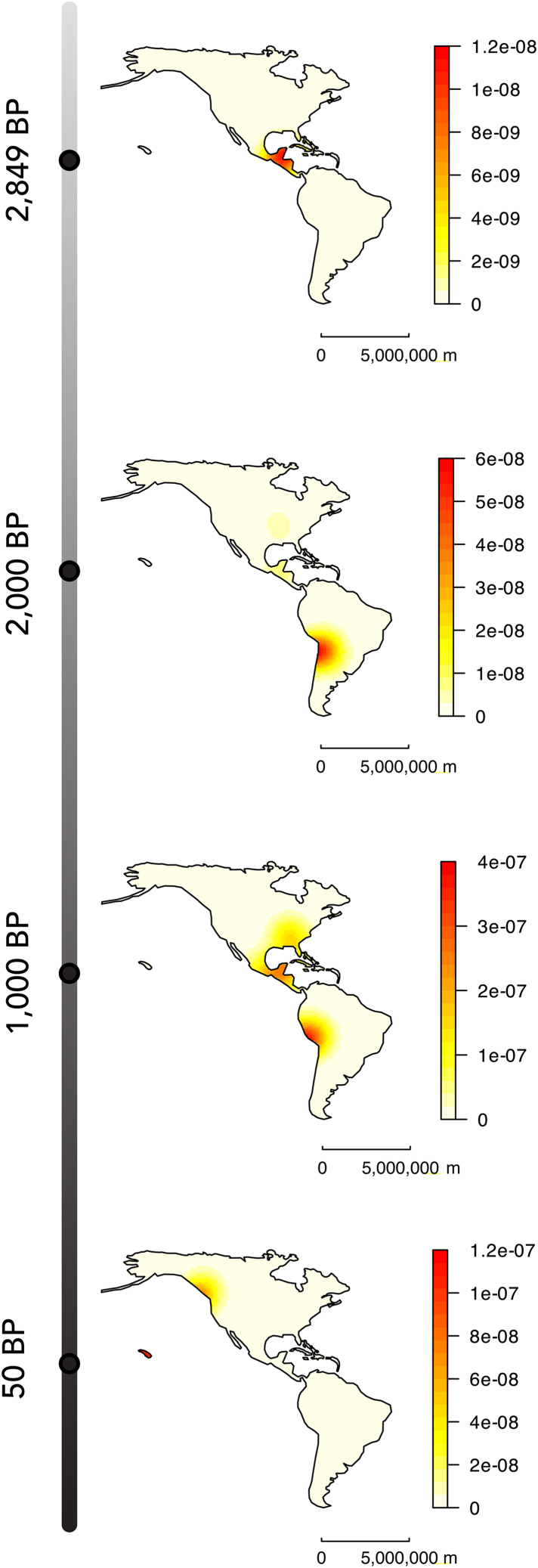
Spatiotemporal kernel density estimation plots showing variation in the density of annular and tabular ICM cases through time in the Americas These plots suggest that the geographic range of ICM extended north and south from Mesoamerica over time. Only major land masses are shown. Scale bar represents 5,000,000 m or 5,000 km.

ICM practice was prevalent in high densities across Mesoamerica by 2,849 BP (Figure 2). A localized high-density cluster of ICM cases is present near what is now the Peru-Chile border on the west coast of South America by 2,000 BP, alongside clusters of low to moderate density in Mesoamerica and North America. The Mesoamerican cluster increases in density to become a high-density hotspot by 1,000 BP, while the hotspot in North America increases to a moderate density. The hotspot in South America remains at high density but shifts north to center on what is now the south coast of Peru. ICM cases also appear along the northern and western coasts of the Gulf of Mexico at low densities, by 1,000 BP, creating a contiguous area of ICM practice that links the Mesoamerican and North American hotspots. ICM is present at moderate densities along the northwest coast of North America and in high densities in Hawaii by 50 BP.

### Empirical Bayesian kriging

**Figure 3:**
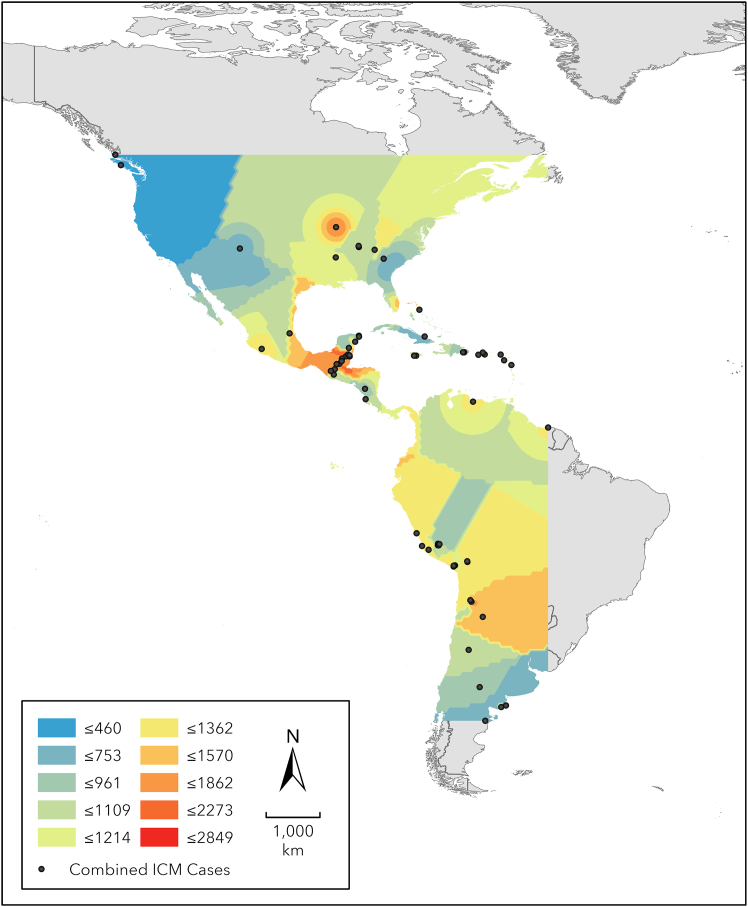
Empirical Bayesian kriging prediction surfaces showing the estimated age of occurrence (in years BP) for annular and tabular ICM cases in the Americas ICM practice was prevalent in Mesoamerica by ca. 2, 273 BP, in Patagonia by ca. 753 BP, and in the Vancouver region of North America by ca. 460 BP. Scale bar represents 1,000 km.

The estimated dates below are extrapolated from empirical Bayesian Kriging (EBK) prediction surfaces (Figures 3, S5, and S6) and represent the latest occurrence predicted for ICM in that area.

The combined ICM prediction surface (Figure 3) suggests that ICM practice was prevalent in Mesoamerica by ca. 2,273 BP. It is then observed along the western margin of the Gulf of Mexico and in southern Mexico, northern Honduras, and isolated areas in the center of North America by 1,862 BP and in eastern Mexico and the Bahamas by ca. 1,570 BP. ICM practice was also prevalent in the center of South America by ca. 1,570 BP, as well as the northwestern portion of South America, isolated hotspots along the northern coast of South America, and the west coast of Mexico by ca. 1,362 BP. ICM was prevalent across the entirety of the center of North America, northeast North America, in the Lesser Antilles and eastern portion of the Greater Antilles, and the remainder of the north coast of South America between ca. 2,214 and 1,109 BP. ICM practice was also observed in southern South America and the western portion of the Greater Antilles by ca. 753 BP and the northwest of North America by ca. 460 BP.

Tabular ICM was first observed (i.e., based on our prediction surfaces) in North America and Mesoamerica by ca. 1,915 BP and in central South America by ca. 1,621 BP. It was prevalent in southern Mexico, the northwest portion of South America, and the north coast of South America by 1,403 BP, the northern border of the Gulf of Mexico and the eastern half of North America by 1,122 BP, and the western half of North America and northern South America by ca. 960 BP. Tabular ICM was also present in Patagonia, the Colorado Plateau, and the central Caribbean by ca. 742 BP (Figure S5).

Annular ICM was present in the Atacama region of western South America and central Bolivia by 1,592 BP and in what is now southwest Peru to the eastern half of North America by 1,036 BP. Annular ICM was then observed in the northwest of North America by 512 BP (Figure S6).

The root-mean-square estimate for all prediction surfaces ranged from 70.16 to 77.72 (Table S3). The average standard errors for each surface ranged from 74.30 to 90.22, with larger errors being associated with “older” predictions and areas lacking spatial observations (Figures S7 and S8). As such, the EBK predictions should be treated with caution.

### Spatial regression analysis

Model residuals obtained by a standard ordinary least squares (OLS) regression exhibited spatial autocorrelation (Table S4). We opted to use spatial error regression based on Lagrange multiplier analysis, which indicated this model was the best fit for our data (Table S5; see Methods S1 for details).

**Figure 4:**
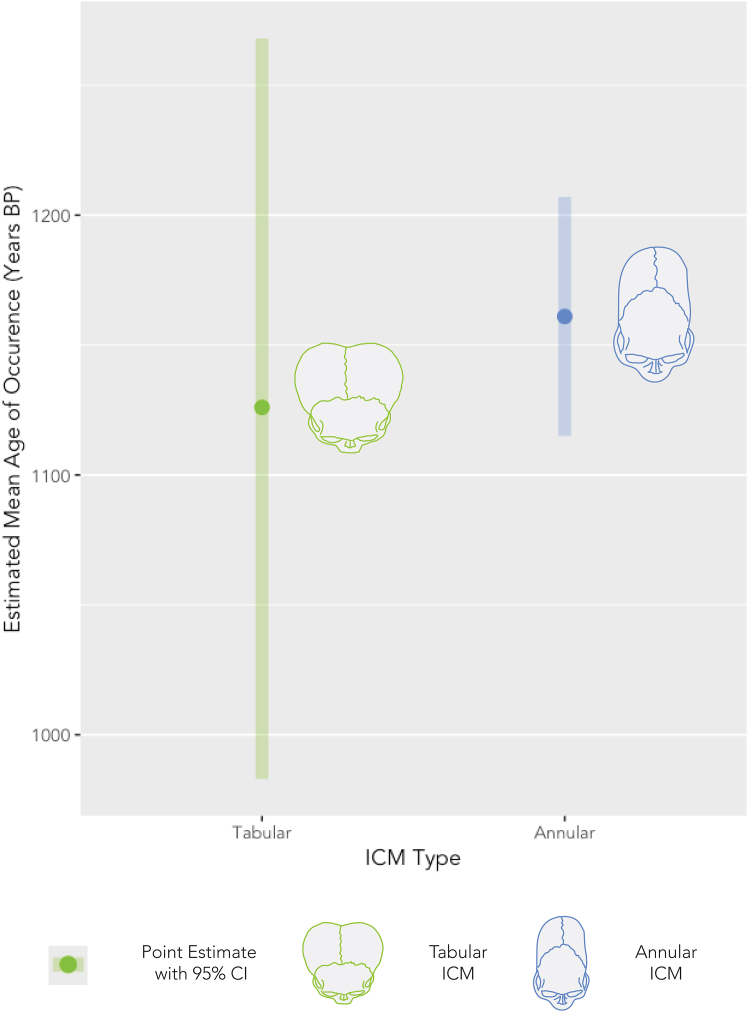
Spatial regression margins plot demonstrating the estimates for the earliest occurrences of ICM in the Americas Point estimates are represented by solid dots, while the bars represent the 95% confidence interval around each estimate. The difference between these estimates is not statistically significant, suggesting that the annular and tabular ICM cases were contemporaneous across this region.

We evaluated the model, Max Age ∼ ICM Type. The small difference (35 years) between the point estimates for the appearance of annular ICM (1,161 BP) and tabular ICM (1,126 BP) was not statistically significantly different (*p* = 0.130), suggesting an almost contemporaneous appearance of annular and tabular ICM practice in the Americas (Figure 4 and Table S6).

Although model diagnostics indicate normality of residuals, the regression estimates should be treated with caution (Figure S9; Tables S7 and S8; and Methods S1).

## Discussion

The deep time perspective on ICM presented here has significant implications for our understanding of diachronic variation in the geographic distribution of ICM practices and the processes underlying this variation. Here, we synthesize our results and outline possible cultural processes driving spatiotemporal change in the occurrence of ICM, with the aim of generating fresh hypotheses for discussion and testing (presented as hypotheses one, two, and three below). Our work is built on the underlying assumption that the spatial distribution of ICM would change over time and that these changes would reflect variation in the underlying stochastic processes producing that distribution.^32^^,^^33^ As ICM occurs in a diverse range of cultural contexts and can be characterized as both a cultural practice perpetuated through cultural transmission and a mechanism for the social reproduction of social structures, beliefs, and norms, we consider a range of sociocultural processes and drivers of change below in favor of a single evolutionary framework. Myriad factors, including human mobility, cultural learning biases,^34^ environmental barriers, interpersonal conflicts, and colonization and forced migration events (e.g., Klaus’s work^35^), can influence the realization of these processes. Further, our EBK and regression results represent estimated ages of occurrence of ICM predicted from the data, rather than empirical observations, and may not correspond directly to dates presented in the literature. The following interpretations should, therefore, be considered with caution.^33^

### Hypothesis one: Spatiotemporal variation in head shaping reflects changes within and between communities

Evaluation of the cross-*K* function showed that the relationships between cases of ICM change at a distance of ca. 500 km (Figures S3 and S4). For cases of ICM that were closer than 500 km, those of annular ICM were significantly more aggregated around tabular cases than expected, whereas at distances greater than 500 km, annular ICM was significantly more dispersed than expected. Recent perspectives of cranial modification, informed by social theory, conceptualize head shaping as a social marker of aspects of identity, including social or ethnic groups, and suggest that it provided a mechanism for responses to social change.^36^ We, therefore, suggest that changing spatiotemporal relationships within and between types of ICM may reflect sociocultural changes within and between communities. For example, modifications are imposed on individuals by family members, and, as such, modified crania could represent a means of enacting power and control over individuals to meet changing social expectations or under conditions of hegemonic acculturation.^1^^,^^2^^,^^37^^,^^38^ These social expectations may, in turn, result in cultural biases that promote the transmission of ICM, such as prestige bias (“copy the famous”) and proximity bias (“imitate those around you”).^39^ Credibility-enhancing displays^40^ could be one mechanism for enhancing and exploiting these biases. Alternatively, studies of head shaping in Europe and South America suggest that cranial modification was used to signal social roles to outsiders as a way of maintaining social identities during periods of change.^1^^,^^2^^,^^9^^,^^10^, This practice could, in part, be explained by schismogenesis, or an individual’s tendency to define themselves in response to others.^41^^,^^42^ This could, in turn, suggest that communities embraced multi-ethnicity, as opposed to hegemonic acculturation, as seen in the case of the south-central Andes around the Tiwanaku state, AD 600–1,000.^38^ Our analyses have suggested several potential scenarios of cultural transmission or innovation that contextual data could test. This includes integration with genetic data that may provide evidence of migration, as reported in female-biased immigration in early Medieval.^43^

### Hypothesis two: Annular modifications represent a derived cultural behavior

The presence of spatial autocorrelation in the maximum age of occurrence of ICM and between ICM types suggests that sites nearer to one another may have adopted ICM behaviors at similar times and that annular ICM was more readily adopted in areas where tabular ICM was already present. That annular ICM tends to appear where tabular cases are present further suggests that annular ICM may be a derived cultural behavior and that existing familiarity with cranial modification behaviors aided the adoption of additional methods of head shaping. Our descriptive and EBK results suggest that annular ICM appeared later and achieved a broad geographic distribution more rapidly than tabular ICM, supporting this supposition. Size and other intrinsic features of populations may have impacted the cultural transmission of this practice.^44^ In addition, the variable adoption of different ICM practices could reflect different social meanings of head shapes; for instance, tabular modifications could reflect embodied social norms and beliefs, while annular modifications could represent fashion, a cultural trait under frequency-dependent transmission.^45^ Social displays, such as cranial modification, may intensify in times of environmental, economic, or social stress, creating the fluctuations seen in ICM use over time. Given the diverse influences on the adoption of ICM behaviors, we do not support generalizing the transmission mechanisms that drove the production of certain spatial patterns across geographic regions. Specific regions may be characterized by distinct cultural evolutionary processes.

### Hypothesis three: Central America and the Caribbean represent independent centers of innovation of ICM

**Figure 5:**
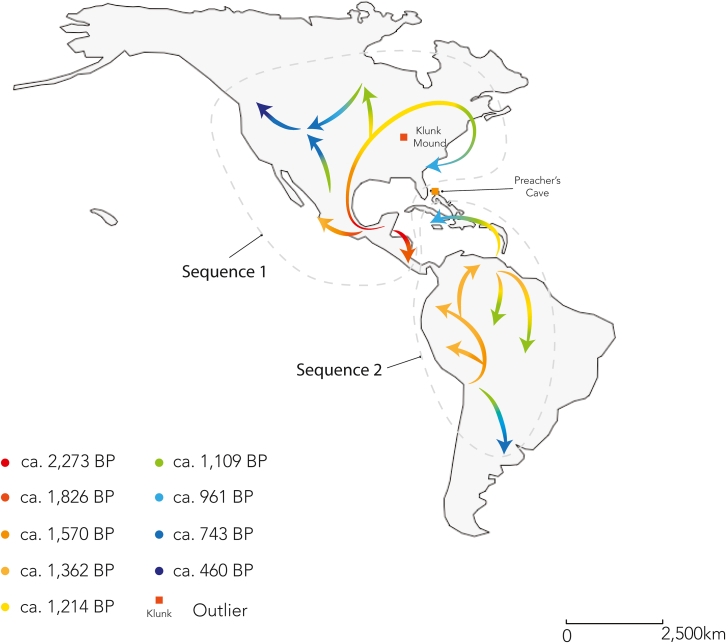
Hypothesized sequences of temporal change in the geographic distribution of ICM, as indicated by our combined ICM EBK prediction surfaces, to be tested individually using specific contextual information in future studies We identify two possible pathways: the first temporal sequence, observed in Mesoamerica and North America, suggests that the first case of ICM occurred in the Yucatan Peninsula of Mesoamerica and that its spatial distribution encompassed the southern portion of Mesoamerica within ca. 400 years. ICM was not observed again in this region for ca. 450 years; however, the geographic range of ICM continued to extend into the eastern portion of North America through this period, reaching the east coast between ca. 1,109 and 961 BP. However, ICM cases (*n* = 27) were also witnessed at Klunk Mound ca. 1,826 BP, suggesting an earlier adoption of ICM in this region. A second sequence, observed in South America and the Caribbean, is separated from the first sequence by spatiotemporal discontinuities in the appearance of ICM in Mesoamerica. This second sequence indicates that ICM was prevalent in west-central South America by ca. 1,570 BP and appeared to shift to the north and east over the following 400 years; ICM was not observed in the very south of South America until ca. 400 years after its initial appearance in central South America. ICM appears to enter the Caribbean from the south at ca. 1,124 BP, although we note the presence of ICM cases (*n* = 2) at Preacher’s Cave in the Bahamas as early as ca. 1,570 BP. Scale bar represents 2,500 km.

Our analyses suggest that an independent center of innovation of tabular ICM occurred in Mesoamerica ca. 3,000 BP (Figure 5). Previous models of the dissemination of ICM suggest the following: (1) ICM reached the Americas either via northeast Asia^28^^,^^29^ or via Polynesian contact with the Americas^8^^,^^13^^,^^25^; and (2) there were independent centers for the development of ICM in Eurasia and the Americas.^30^ Our prediction surfaces show spatial discontinuities in the presence of ICM, suggesting that though ICM was present in Mesoamerica by ca. 3,000 BP and reached northeastern North America at 0–1,000 BP, it was not present in south of Mesoamerica at this time. Given the absence of evidence of cases decreasing in age as they reached the southern latitudes, we do not support the hypothesis that ICM reached the Americas via Asia. Likewise, our data show that the first appearance of ICM in the Americas predates the Austronesian expansion into the eastern Pacific^46^^,^^47^ and the hypothesized Polynesian contact with the Americas,^48^ based on genetic markers of native Americans in early settlers of Rapa Nui (Easter Island). As such, as in several cases of material culture,^49^ similarities between Polynesia and South America predating 1,000 BP are best explained as cultural convergences. As such, our analyses suggest hypotheses that contradict many of the diffusionist ideas on ICM generated throughout the 19^th^ and 20^th^ centuries.

Myriad cultural exchanges occurred between Mesoamerica and South America in pre-Columbian times,^50^ including maize, cacao, tobacco, and potentially coatless dogs,^51^^,^^52^^,^^53^ which supports the possibility of an independent center of development of ICM in South America. Our data also show that the geographic distribution of tabular ICM extends from south to north in South America between ca. 1,500 and 1,100 BP. The lack of geographic spread from north to south out of Mesoamerica suggests that this later occurrence of ICM represents an additional center of innovation. During this period, large scale economic and social changes were witnessed in Chile, including possible migration and population replacement processes that may have shaped the spatiotemporal distribution of ICM.^54^ Recent syntheses of archaeological, ethnohistorical, linguistic, and genetic data suggest that human migration likely followed waterways in northern South America, providing a framework for understanding how cultural exchanges of ICM behavior could have occurred.^55^

Finally, our data suggest that ICM reached the Caribbean through South America (Figure 5). Hofman^56^ discussed sustained interaction networks between the Lesser Antilles and mainland South America, especially the Guianas and the Orinoco region. This provides a strong contextual basis for hypothesizing the transmission of cultural practices like ICM along these networks. Similarly, the many records of ICM of northern Venezuela^57^ and the Arawak expansion into the Caribbean are well established, even though the routes of dispersal remain contested.^58^ Differences in the presence of ICM between islands may relate to different waves of migrations.^59^

Alongside processes of human movement, interaction, and exchange, cultural evolutionary models such as the one by Heinrich,^40^ “credibility-enhancing displays,” may provide useful frameworks for understanding the independent innovation of acts of costly signaling such as ICM.

### Hypothesizing cultural evolutionary processes shaping spatiotemporal distributions of ICM across the Americas

The temporal separation between the first occurrences of ICM in the Americas and those in Asia^29^ speak for the cultural convergence of this trait.^30^ This mirrors the observation that ornamentations are evolutionarily labile traits,^60^^,^^61^ particularly in the context of cultural evolution where they are prone to convergence.

Regional studies of ICM^25^^,^^26^^,^^27^^,^^62^^,^^63^^,^^64^ have indicated great cultural diversity in both the social significance of cranial modification practices and the cultural biases influencing the cultural transmission of information related to this practice. For example, whether head shaping is used as a visual marker of social identity varies according to culture and region^8^; in the Pampa-Patagonia region of Argentina during the late Holocene, head shape in some cases related to “fashion” more than to cultural differentiation (Serna et al.^65^). The frequency of ICM in some regions also does not equate to uniformity in modification types. For example, contrasting types were present in Tiwanaku in the southern Andes ca. AD 500–1,100,^66^ which presents further challenges in understanding cultural transmission and innovation of the practice. The multiple developments of ICM are mirrored in the different social contexts and modes in which this practice has been recorded.^10^^,^^38^ The mosaic nature of the distribution in space and time of ICM supports these assumptions. The temporal persistence of ICM may be linked to its transmission fidelity, with the latter established as being positively related to trait longevity,^67^ because head shaping takes months to achieve, which allows for highly accurate transmission of information. As is typical for archaeology, more data are available for periods closer to the present.

ICM was surely embedded in other belief practices and costly acts,^40^ and deciphering the diversity of its significance across time and space will require, for each record, assessment of the relationship with factors such as diet, sex, and violence (e.g., Okumura’s work^68^) and information on material culture and geographic origins.^1^^,^^9^^,^^69^

### Limitations of the study

Spatial patterns have often been viewed as proxies for sociocultural processes in studies of past human populations. However, little is known about the behaviors, movements, and activities of these underlying populations; thus, all results, particularly those with a visual component (e.g., maps), must be considered conservatively as evidence of presence or “exposure” only to avoid oversimplifying human behaviors.^6^^,^^70^

The spatial error term was still significant in our final regression model, suggesting that there is a spatially correlated predictor not accounted for in our model.^71^ Uncontrolled spatial error can make estimates inefficient (i.e., having a large variance) and produce incorrect standard errors.^72^ Our regression results should, therefore, be interpreted with caution. We estimated the locations of archaeological sites by using Google Earth, where cases were located only to a region, and co-ordinates were assigned as the center of that region. This procedure may result in an underestimation of ICM prevalence at the local scale.^71^

Our analysis assumes that the oldest dates for a site represent the earliest occurrence of ICM at that location. However, the earliest human experimentation with ICM may not have been occurring at a scale or duration that is archaeologically detectable today. Our research also did not differentiate between intentional and non-intentional cranial modifications, which may have predated the occurrence of intentional practice. This conflation of ICM types may artificially inflate the true prevalence of ICM behavior and distort spatial representations of this practice. Furthermore, our study relies on skeletal evidence of ICM, which is subject to preservation and sampling biases. The preservation of human remains has been shaped by myriad factors, including the age and sex of an individual, mortuary practices, and the burial environment,^73^^,^^74^ while sampling biases are shaped by archaeological survey, excavation, and sampling methods.^74^^,^^75^ It is, therefore, likely that our approach underestimates the antiquity of ICM in the Americas.

There is a strong need for the addition of social, economic, and environmental (including topographic) data and consideration of a broader range of ICM types in future iterations of broad-scale analyses to gain insights into the drivers of the adoption of ICM. This would, in turn, support the investigation of more advanced hypotheses, such as the impact of geographical barriers on the cultural transmission of ICM. For instance, Dingwall^13^ and Dembo and Imbelloni^4^ suggested that water courses seem to coincide with the paths of potential transmission of ICM practices, an observation that could be tested with additional variables. Similarly, a close examination of the regional variations in cultural transmission bias(es) associated with ICM will provide fundamental insights into the dynamics of cultural evolution.

## Resource availability

### Lead contact

Requests for further information and resources should be directed to and will be fulfilled by the lead contact, Marcelo Sánchez-Villagra (m.sanchez@pim.uzh.ch).

### Materials availability

This study did not generate new unique reagents.

### Data and code availability

• The Global ICM data have been deposited at Zenodo and are publicly available as of the date of publication at https://doi.org/10.5281/zenodo.14499026.
• All original code has been deposited at Zenodo and is publicly available as of the date of publication at https://doi.org/10.5281/zenodo.14499026.
• Any additional information required to reanalyze the data reported in this paper is available from the lead contact upon request.

## Acknowledgments

This work was supported by 10.13039/501100001711Swiss National Science Foundation (10.13039/501100001711SNF) project number 208545 to M.R.S.-V. and Analía Forasiepi. L.A.B.W. is supported by the Discovery Program of the 10.13039/501100000923Australian Research Council (FT200100822). G.R.-d.L. is supported by the 10.13039/501100004837Spanish Ministry of Science and Innovation and the 10.13039/501100004895European Social Fund (grant RYC2024-051368-I funded by MICIU/AEI/10.13039/501100011033 and by ESF+). We thank the anonymous reviewers for their suggestions, which helped improve this manuscript.

## Author contributions

M.R.S.-V. and L.A.B.W. conceptualized the project: S.M.W. and L.A.B.W. developed the methodology; S.M.W. conducted the analyses; C.R., G.R.-d.L., and S.L. built the database; M.R.S.-V. and L.A.B.W. acquired funding; S.M.W., M.R.S.-V., and L.A.B.W. wrote the original draft. All authors have reviewed and edited the manuscript.

## Declaration of interests

The authors declare no competing interests.

## STAR★Methods

### Key resources table

**Table:** 

REAGENT or RESOURCE	SOURCE	IDENTIFIER
**Deposited data**		

Americas Only ICM Data	This study	https://doi.org/10.5281/zenodo.14499026
Data for Margins Plot	This study	https://doi.org/10.5281/zenodo.14499026

**Software and algorithms**		

ImageJ	Schneider et al.^76^	https://imagej.nih.gov/ij/
ARC GIS Pro	ESRI Inc.^77^	https://www.esri.com/en-us/arcgis/products/arcgis-pro/overview
RStudio	POSIT Team^78^	https://posit.co/download/rstudio-desktop/
R Code	This study	https://doi.org/10.5281/zenodo.14499026

### Method details

#### Ethical considerations

This section outlines the steps taken to ensure the ethicality of the present research. An outline of our positionality and the broader ethical context of this work is presented in Methods S2.

The present study is based exclusively on secondary data derived from previously published, peer-reviewed sources and no new analyses were conducted on physical human remains as part of this project. As our final dataset comprised over 2000 individuals of unknown identity spanning 21 countries and 3000 years of human history, we were unable to consult with the communities of origin of our participants. Further, we note that ethnic affiliations cannot easily be inferred for archaeological individuals (defined here as ≥100 years old) or used to identify descendant communities due to the sociocultural complexity of identity and theoretical, political, and methodological challenges in linking identity with language, biology, or archaeological materials.^79^^,^^80^^,^^81^ We therefore sought guidance from our institutional Human Research Ethics Committee (HREC) per Squires et al.,^82^ and were advised our work did not require ethical approvals. To formalise this advice, we applied for an ethical exemption, which was subsequently granted on the grounds that the research (1) uses deidentified, pre-existing data already in the public record; and (2) has a negligible risk of harm to the research participants, defined as both the skeletal individuals and their descendant communities (Australian National University HREC Protocol H/2025/0582).

However, we acknowledge that ethical responsibility does not end with primary data collection and acknowledge that there is potential for our study to cause discomfort to descendant groups. To mitigate possible discomfort to living communities, we have followed current recommendations for the ethical use and presentation of data in Biological Anthropology. These recommendations emphasise respectful, personhood-centred representations of the deceased,^83^^,^^84^^,^^85^^,^^86^^,^^87^ avoidance of sensationalism in research presentation,^88^ and adoption of cultural sensitivity.^82^ In this way, our study contributes to a growing body of research that seeks to treat ancestral remains not just as scientific “objects”, but people deserving of dignity.^85^^,^^87^^,^^89^ Our experience further highlights the need for detailed guidelines for the care of skeletal individuals and their associated data.^89^

#### Literature search

To create a global database of ICM cases across time and space for analysis, we conducted a multi-year, traditional literature search of online publications sourced from English and Spanish language platforms (search terms provided in Table S9). These platforms included online journals, publication databases, and university libraries. All published cases of cranial modification that were both archaeological and identified as intentional were curated in the database, with different cases of ICM within a site recorded as separate individuals.

#### Construction of the global database

Raw variables extracted from each source were: ICM type, sex, dates, and spatial location for each individual (see Table S10 for further details). Spatial locations, in the form of geographic coordinates, were obtained from publications and converted to decimal degrees where required. Where geographic co-ordinates were not provided in a publication, they were approximated by locating the site using Google Maps. Where cases were published as regional or multi-site syntheses and it was not possible to determine the specific location of each case, individual locations were estimated as the centre of the study region. Lastly, we recorded the maximum (e.g., oldest) age of each site in years BP to approximate the earliest date for the occurrence of ICM behaviours for each location. Where individual cases of ICM could be identified to a specific period within a site chronology, they were assigned the maximum dates for this period. All relative dates (e.g., Classic period, Postclassic period) were converted into absolute age ranges based on currently accepted regional chronologies (see Table S10 for further detail). All chronologies were converted into years before present (BP) for analysis.

The resulting database comprised 2109 ICM cases across all world regions. Here, we present an analysis of the Americas portion of the database only (Table S11). This region was selected for analysis as it represents the largest and most complete section of our data.

#### Database cleaning and preparation of the Americas only dataset

Additional processing was undertaken using RStudio (Version 2023.9.1.494^78^) to streamline the dataset for spatial analysis (see Figure S2 for overview). All individuals with an unspecified (n=4) or tumpline (n=35) modification type were excluded from analysis due to small sample sizes, and all ICM types were collapsed into the base categories “Tabular” and “Annular”^4^ to increase the accuracy of ICM classifications and increase sample sizes for analysis.

As spatial analysis does not support the presence of duplicated co-ordinates, all coordinates were “jittered” by adding 0.0000001 (∼10m) to the existing latitude and longitude values to create unique coordinates for all individuals. All remaining duplicated coordinates (n=25), sites falling outside of the Americas (n=326) were identified and excluded from analysis, resulting in a total sample size of 1722 cases of ICM for analysis.

To obtain coordinates for the spatial analysis window, we exported a world map with a pseudo-Plate Carée projection and Cartesian grid from Esri ArcGIS Pro (Version 3.0.3^77^) and imported into ImageJ (Version 1.53k^76^). The y-axis of the image was inverted to place the coordinate origin in the bottom left of the image and the image units were set to decimal degrees. The image scale was established by using the line tool to place a straight line between map graticules ten decimal degrees apart on the imported image. All parallels and meridians in pseudo-Plate Carée projections form perfect squares and remain equidistant at all locations on an image. As such, the scale of the image is uniform and one scale can be applied.

To obtain a set of coordinates defining the boundary of each major land mass, the point tool was used to place points anticlockwise around the boundaries of each region. The centroid of each point was obtained using the measure tool, yielding “*xy*” co-ordinates for each point in decimal degrees. Land boundary and ICM case coordinates were projected into the Plate Carée Projected Coordinate System (datum = WGS 1984, ellipsoid = WGS 1984) to support spatial statistical analysis, which requires Euclidean distances. The Plate Carée projection was selected as it preserves distances, ensuring distance-based analyses, such as those employed in this study, are reliable.^90^^,^^91^ However, Plate Carée projection is known to distort the shape, angles, and areas of projected features, with distortions being most severe close to the poles.^90^^,^^91^ As our data fall outside the polar latitudes, we anticipate the impacts of these distortions will be minimal. Separate point pattern objects were produced for each ICM type (Annular, Tabular, and Combined, i.e., both Annular and Tabular).

### Quantification and statistical analysis

Spatial analysis was split into data exploration, visualisation, and modeling phases as per standard spatial analytical procedure presented in Bailey and Gatrell.^92^ Where test parameters are not specifically noted, the programme defaults were used.

#### Spatial data exploration

We extracted an initial set of descriptive statistics, comprising the mean, maximum, minimum, and standard deviation of the maximum age of occurrence of ICM for each ICM type to capture the central tendency and dispersion of the data.

Spatial data exploration typically follows an inductive approach, where patterns are observed from data and used to generate hypotheses.^93^ As such, it is typically performed without making pre-existing assumptions regarding patterns on, and relationships between spatial data.^94^ The types of patterns and relationships identified in spatial data then inform the selection of statistical modeling approaches in subsequent phases of analysis.^95^ Commonly observed patterns in spatial data include spatial autocorrelation and non-stationarity in both geographic location and case characteristics.^96^^,^^97^ The presence of these trends in spatial data violates the assumptions of non-spatial statistics, leading to false positives and inaccurate confidence intervals.^96^^,^^97^ Spatial autocorrelation, or dependence, occurs when an attribute associated with one location is influenced by an attribute associated with a neighbouring location, causing attributes nearer to each other to be more similar to one another than those further apart.^95^^,^^97^ Non-stationarity, or heterogeneity, of spatial data occurs when this relationship between attributes changes across space.^97^ To inform the selection of approaches for the modelling phase of our analysis, we first performed Moran’s *I* tests to explore whether spatial autocorrelation was present in the maximum ages of occurrence for ICM.^95^^,^^98^^,^^99^ We assumed spatial randomness and employed a row-normalised inverse distance spatial weights matrix to simulate distance-based decay in spatial dependence.^100^ To test for the presence of spatial autocorrelation between different ICM types, we evaluated the inhomogeneous cross-type *K*-function.^101^ The standard *K*-function is commonly employed to assess for spatial dependence (i.e., clustering) in the locations of points of a single type.^95^^,^^100^^,^^102^ The inhomogeneous, cross-type generalisation of this function estimates the “number of points of type *j* lying within a distance *r* of a typical point of type *i,* standardised by dividing by the intensity of points of type *j*.” 101: 594. It therefore adjusts for differences in sample size and variations in the density of spatial observations, and considers spatial dependence between points of different types, making it suitable for heterogenous datasets.^101^^,^^103^ The inhomogeneous cross-type *K*-function test was conducted in a pointwise fashion, specifying Ripley’s isotopic edge correction factor.^104^ Tolerance envelopes (95%) were calculated from 39 simulations of CSR.^103^

#### Spatial data visualisation

We used spatiotemporal kernel density estimation (stKDE) and empirical Bayesian kriging (EBK) to visualise broad spatiotemporal trends in the occurrence of ICM where these were identified during spatial data exploration.

#### Spatiotemporal kernel density estimation (StKDE)

StKDE is a non-parametric smoothing technique for estimating the density of continuous space-time data and is used to visualise the aggregation of spatial observations through space-time.^105^^,^^106^^,^^107^ Standard kernel density estimation involves placing a 2D probability density function, or “kernel”, over each point in a pattern. These kernels can be conceptualised as bell-shaped. The spread of each kernel is controlled by a bandwidth, which specifies the kernel’s radius. Larger bandwidths smooth out variation in density, while smaller bandwidths detect smaller fluctuations in density.^105^ The overlapping densities are then summed for each node in a grid laid over the point pattern (e.g., pixels) producing a probability density estimate for each node.^100^^,^^105^ These densities are then plotted to create a “heat map” showing areas of high and low density. In the spatiotemporal extension of this technique, the density surface is smoothed via a 3D kernel function, which is the product of a 2D “spatial” (x, y) kernel and a 1D “temporal” (z) kernel. We chose Gaussian kernels for both the spatial and temporal dimensions, as the mathematical and theoretical properties of this kernel are robust to data heterogeneity and areas without spatial observations.^105^ We then calculated fixed, isotropic, oversmoothing spatial and temporal bandwidths from our data using the bandwidth selecter “OS.spattemp”.^108^ This command produces two numeric values that specify the optimal bandwidths for the spatial and temporal domains of our data in metres and years, respectively. Evaluation of the inhomogeneous *K*-function suggests that relationships between ICM cases varied at the regional level (Figures S2 and S3). Our spatial bandwidth (*h* = 686km) is therefore sized to detect variations in ICM case density at the regional level. To enable the extraction of time slices at yearly intervals, we rounded the maximum and minimum age of ICM occurrence for each subset up and down to the nearest 1000 respectively. We then estimated the annual density of ICM cases every using the default time resolution and spatial and temporal edge correction settings. Density estimates were multiplied by one billion to support plot accessibility. “Time slices” depicting the joint density of ICM cases approximately every 1000 years were extracted and ordinated by ICM type to visualise the dispersion of ICM behaviours over time and space. We chose the joint density estimate to facilitate comparison of changes in density over time.^109^ Although density is proportional to intensity (the number of points per area),^110^ we describe our results in terms of density to avoid making assumptions regarding behavioural intensity. High density areas are identified as those estimates at or above the 75^th^ percentile for densities in that region at a particular time. We describe ICM densities in relative terms (e.g., higher, lower) to enable comparison of results at varying spatiotemporal scales.

#### Empirical Bayesian Kriging (EBK)

The ultimate sample of skeletal remains recovered through archaeological excavation, as documented in our database of ICM cases, is shaped by a myriad of factors.^74^ To predict the spread of ICM behaviours in undersampled subregions, notably the eastern portion of South America, we used Empirical Bayesian Kriging (EBK), which is a probabilistic method of spatial interpolation that predicts data where spatial observations are missing.^111^

Kriging consists of two stages: 1) semivariogram creation and model fitting and 2) and prediction via a kriging system. Semivariograms summarise the spatial relationships between locations and are used to predict data where observations are missing.^112^^,^^113^ In Empirical Bayesian Kriging (EBK), several semivariograms are constructed for each observation, with each model being estimated using data simulated from the model before and thereby accounting for the error introduced into predictions by the semivariogram itself.^111^ Weights are produced from the semivariogram using Bayes’s rule, which assesses the likelihood of a particular model producing the data observed, and are used in turn to make predictions in areas of missing data.^111^ Predictions are produced through intrinsic random function kriging (IRFK) using a restricted maximum likelihood estimator.^111^^,^^114^

We performed EBK using the EBK tool in the Geostatistical Analyst Toolbox in ArcGIS Pro (v. 3.0.3^77^). Empirical Bayesian Kriging was chosen as it is robust to small, non-stationary datasets.^111^ The distribution of each dataset was assessed using histograms and found to be non-normal. As such, the data were transformed within the EBK tool using via multiplicative skewing with an empirical base distribution. We chose a K-Bessel semivariogram model and increased the number of simulated variograms produced to minimise standard errors.^114^ We increased the maximum points in each subset (125-150) and subset overlap factor (2-3) to reduce noise in the prediction surface (Table S3). All neighbourhood settings were left as default: full or “one sector” standard circular neighbourhood containing a maximum of 15 neighbours and minimum of 10 neighbours, no rotation, and with search radii (automatically calculated based on the area of the region being analysed) of approximately 4,000km (Table S3). The “fit” of each prediction surface was assessed using the root mean squared error and average standard error.

#### Spatial modeling

The human brain recognises patterns and may perceive these in randomly organised data and vice versa.^33^^,^^93^ To confirm the spatial patterns identified through the visual assessment of our ICM data and to test whether these patterns were statistically significant, we undertook spatial modelling using spatial autoregression. Spatial autoregressive approaches account for the spatial structure, including autocorrelation and heterogeneity, present in spatial data.^100^

Following Anselin,^115^ we performed a non-spatial linear regression using an ordinary least squares (OLS) estimator. We then used Moran’s *I* to test for spatial autocorrelation in the model residuals, as above.^97^^,^^116^^,^^117^ Where Moran’s *I* suggested spatial autocorrelation of the residuals, we used Lagrange Multiplier tests to determine which type of spatial regression would provide the best fit for our data relative to an OLS model.^116^^,^^117^^,^^118^^,^^119^ We considered both spatial error models, which are used to correct for autocorrelation in a model’s error terms,^117^ and spatial lag models, which control for spatial autocorrelation in the dependent variable, as alternatives to OLS regression and calculated both standard and robust linear model statistics for these models using an inverse-distance spatial weights matrix.^116^ We assessed the p-values for the standard tests and chose the test with the most statistical significance for use (i.e., smallest p-value) as per Anselin.^117^ Where both tests were statistically significant, we assessed their robust forms and retained the most statistically significant test for use.^116^^,^^117^ Where both robust tests were significant, we chose the test with the higher test statistic.^120^

We then performed maximum likelihood estimation of a spatial autoregressive error model^116^^,^^121^^,^^122^^,^^123^^,^^124^ using an inverse-distance spatial weights matrix. The response variable was the maximum age of occurrence of ICM behaviour in years BP, and predictor variable was ICM type. Likelihood ratio tests were used to assess whether spatial error had been adequately accounted for within the model, with statistically significant values interpreted as representing remaining uncontrolled error. Lastly, we extracted the summary data for our model and completed a series of diagnostic tests to assess whether the final model chosen met the statistical assumptions of linear regression. These tests included residual-vs-fitted-values plots and studentised Breusch-Pagan tests, which were used to assess the data for heteroskedasticity, and histograms of the residuals to assess whether the data followed a normal distribution.

## Supplemental information

Document S1. Figures S1–S9, Tables S1–S9 and S11, and Methods S1 and S2

Table S10. Variables extracted from data sources retrieved from the literature search, related to STAR Methods, Construction of the Global Database
