# Supplementary material for: Intentional cranial modifications in the Americas: The temporal and spatial patterns of potential transmissions and cultural innovations
Source: iScience. 2026 Apr 8;29(5):115643. doi: 10.1016/j.isci.2026.115643 (PMC13141071; doi:10.1016/j.isci.2026.115643)
Supplement: Document S1. Figures S1–S9, Tables S1–S9 and S11, and Methods S1 and S2 [file mmc1.pdf]

iScience, Volume *29*

Supplemental information

Intentional cranial modifications in the Americas: The temporal and spatial patterns of potential transmissions and cultural innovations

Stacey M. Ward, Marcelo R. Sánchez-Villagra, Caitlin Raymond, Gizéh Rangel-de Lazaro, Sinéad Lynch, and Laura A.B. Wilson

CONTENTS

Methods S1. Description of model assessment methods, related to Results: Spatial Regression Analysis.
Methods S2. Researcher positionality and ethical context of this research, related to Methods: Ethical Considerations.

Figure S1. Histogram plots for Americas annual and tabular datasets, related to Results: Descriptive Analysis.
Figure S2. Data Analysis workflow, related to Results: Descriptive Analysis.
Figure S3. Inhomogeneous *K*-function plots for Americas combined, annular, and tabular datasets, related to Results: Assessment of Spatial Autocorrelation and Non-Stationarity.
Figure S4. Inhomogeneous cross-*K* function plots for the Americas dataset, related to Results: Assessment of Spatial Autocorrelation and Non-Stationarity.
Figure S5. Empirical Bayesian Kriging prediction surface for tabular ICM in the Americas, related to Results: Empirical Bayesian Kriging.
Figure S6. Empirical Bayesian Kriging prediction surface for annular ICM in the Americas, related to Results: Empirical Bayesian Kriging.
Figure S7. Empirical Bayesian Kriging standard error surface for tabular ICM in the Americas, related to Results: Empirical Bayesian Kriging.
Figure S8. Empirical Bayesian Kriging standard error surface for annular ICM in the Americas, related to Results: Empirical Bayesian Kriging.
Figure S9. Spatial simultaneous autoregressive error model diagnostics, related to Results: Spatial Regression Analysis.

Table S1. Descriptive statistics of the sample used in this study, related to Results: Descriptive Analysis.
Table S2. Moran's *I* tests for autocorrelation in the variable "maximum age of ICM occurrence" (years BP), related to Results: Assessment of Spatial Autocorrelation and Non-Stationarity.
Table S3. Prediction parameters and Root Mean Squared Error for the Empirical Bayesian Kriging prediction surfaces, related to Results: Empirical Bayesian Kriging.
Table S4. Moran's *I* tests for autocorrelation in OLS model residuals, related to Results: Spatial Regression Analysis.
Table S5. Lagrange Multiplier tests of spatial dependence in linear models, related to Results: Spatial Regression Analysis.
Table S6. Spatial simultaneous autoregressive error model output, related to Results: Spatial Regression Analysis.
Table S7. Breusch-Pagan Tests for Heteroscedasticity in Spatial Error Model Residuals, related to Results: Spatial Regression Analysis.
Table S8. Likelihood Ratio Test Results for Spatial Error Regression Models, related to Results: Spatial Regression Analysis.
Table S9. Search terms used in the literature review of Intentional Cranial Modification cases, related to STAR Methods: Literature Search and Ethical Considerations.
Table S10. Provided as separate document due to size constraints, please see Table S10.
Table S11. List of localities included in study region, related to STAR Methods: Construction of the Global Database.

Methods S1: Model Assessment, related to Results: Spatial Regression Analysis.

Moran's I confirmed that there was positive spatial autocorrelation in the OLS residuals ($I = 0.95$, $Z = 99.3$, $p<0.001$) (Table S4), indicating that ICM cases of similar ages tend to cluster in our dataset and that the use of a spatial regression model was justified[1,2]. We used standard Lagrange Multiplier (LM) tests to assess which type of spatial regression model would provide the best fit for our data and found that both the lag and error models were statistically significant ($p<0.001$) (Table S5). We then assessed the robust LM for each model, and this test indicated that the spatial error model provided the best fit ($p<0.001$) for all datasets except for Europe and West Asia, where the spatial lag model featured the smallest p-value ($p = 0.18$). As there was minimal difference in the statistical significance of the robust test results for the lag and error models ($p = 0.18$ cf. 0.68 respectively) for Europe and West Asia, we opted to use a spatial error model for ease of interpretation.

Likelihood ratio tests indicated that the spatial error term (λ, lambda) was significant across all models ($p<0.001$), suggesting that the spatial disturbances have not been fully accounted for in our model (Table S8). This generally indicates that spatial autocorrelation in the error terms is a result of an unknown process not accounted for by the model, and that the model is therefore mis-specified [1].

Methods S2: Researcher positionality and ethical context of this research.

At the time of writing this manuscript, the authors comprised a mixture of early-, middle-, and later-career stage researchers from Australasia, Europe, and the Americas. As descendants of colonising powers and settler populations in these regions, we acknowledge our privilege and ongoing responsibility for redressing ongoing structural violence against marginalised peoples. As researchers, we seek to balance the advancement of knowledge with respect for both Ancestral remains and descendant communities.

Respectful care of archaeological human remains has become a core area of consideration in bioarchaeology in recent years[3-8]. Current ethical perspectives advocate for the recognition of the personhood of human remains, repatriation of unethically obtained ancestors, and for the foregrounding of descendant groups in decision-making regarding ancestral remains to address ongoing issues of structural violence in bioarchaeology research[6,7,9].

However, recent publications [10-13] have highlighted that current principles for ethical best practice do not extend to the creation, curation, and use of skeletal data. Uncritical data use may perpetuate structural violence against marginalised and minoritised groups through decontextualising and dehumanising skeletal individuals and promoting unethical, extractive practices for data creation and use [10-14]. These issues can be mitigated through the adoption of Indigenous data governance principles, which emphasise that Indigenous sovereignty and autonomy over ancestral remains must extend to data created through observation of these remains [14,15]. Specifically, descendant communities should have full control over the collection, storage, and use of this data [11]. The recently developed CARE framework [15] provides one pathway for recentering decision-making with descendant communities. Obtaining informed consent for research is critical to supporting this recentering but represents a key challenge in analyses of archaeological human remains of unknown provenance or cultural affiliation [5,16].

Table S1. Summary of descriptive statistics for Intentional Cranial Modification (ICM) cases (N) used in this study. Detailing the type of ICM, its regional distribution, and mean, max, min and SD values, which are provided in years Before Present (BP). Related to Results: Descriptive Analysis.

Region	% of Total Sample	ICM type	N	% of Regional Sample	Mean	Max	Min	SD
Americas	85.1	Combined	1722	100.0	1114.0	2849.0	50.0	505.0
		Annular	348	20.2	906.0	2050.0	150.0	429.0
		Tabular	1374	79.8	1167.0	2849.0	50.0	509.0

Table S2. Moran's Index tests for autocorrelation in the variable "maximum age of ICM occurrence (years BP)". Related to Results: Assessment of Spatial Autocorrelation and Non-Stationarity.

Region	ICM Type	Moran's Index	Expected	SD	Variance	*p*
Americas	Combined	0.959	-0.0006	97.85	0.0001	<0.001
	Annular	0.976	-0.0029	46.25	0.0004	<0.001
	Tabular	0.955	-0.0007	84.54	0.0001	<0.001

Table S3. Prediction parameters for the Empirical Bayesian Kriging prediction surfaces, detailing Root Mean Squared Error (RMSE), average squared error (SE). Parameters are: max neighbours - the maximum number of neighbours that will be used to estimate the value at the unknown location; min. neighbours - the minimum number of neighbours that will be used to estimate the value at the unknown location; sector type – the geometry of the neighbourhood, where 1 sector is an ellipse and more than 1 sector describes division of an ellipse into multiple sectors; Angle – the angle of rotation of the axis (circle) or semi-major axis (ellipse) of the moving window; Radius – the length of the radius of the search circle. Related to Results: Empirical Bayesian Kriging.

Parameter	Americas Combined	Americas Annular	Americas Tabular
Number of observations			
Data transformation type	Empirical	Empirical	Empirical
Semivariogram model type	K-Bessel	K-Bessel	K-Bessel
Max. number points local model	150	125	125
Local model area overlap factor	3	2	2
Number simulated variograms	150	150	150
Search neighbourhood	Std Circ	Std Circ	Std Circ
Max. neighbours	15	15	15
Min. neighbours	10	10	10
Sector type	1 sector	1 sector	1 sector
Angle	0	0	0
Radius	3,859,456	3,859,456	3,859,456
**RMSE**	70.16	77.72	74.26
**Average SE**	74.30	77.30	90.22

Table S4. Moran's I tests for autocorrelation in the OLS (ordinary least squares) model residuals, describing the OLS model assessed in this study. Related to Results: Spatial Regression Analysis.

OLS model	Moran's Index	Expected	Standard Deviate	Variance	*p*
Americas OLS 1 (Max Age ~ ICM Type)	0.95	-0.001	97.59	0.0001	<0.001

Table S5. Lagrange Multiplier tests of spatial dependence in linear models. Methods are Lagrange Multiplier error (LMerr), Lagrange Multiplier lag (LMlag), Robust Lagrange Multiplier error (RLMerr), and Robust Lagrange Multiplier Lag (RLMLag). Related to Results: Spatial Regression Analysis.

Method	Statistic	Americas OLS 1
LMerr	LM	9380.50
	p	0.00
LMlag	LM	9325.60
	p	0.00
RLMerr	LM	64.36
	p	0.00
RLMLag	LM	9.53
	p	0.00

Table S6. Spatial simultaneous autoregressive error model outputs for the 'Americas SE1' model. Related to Results: Spatial Regression Analysis.

Model	Variable	Coefficients	SE	z-value	p	LL CI	UL CI	Wald Test	AIC
Americas SE1 (Max Age ~ ICM Type)	Lambda	0.97	0.00	350.09	<0.001	0.96	0.98	<0.001	20834
	Intercept	1126	72.83	15.45	<0.001	982.79	1268.26		
	Annular	35	23.32	1.51	0.130	-10.43	81.00		

Table S7. Breusch-Pagan Tests for Heteroscedasticity in Spatial Error Model Residuals. Related to Results: Spatial Regression Analysis.

Model	BP Statistic	DF	*p*
Americas SE1 (Max Age ~ ICM Type)	0.023817	1	0.877

BP Statistic = Breusch-Pagan Test Statistic, DF = Degrees of Freedom

Table S8. Likelihood Ratio Test Results for Spatial Error Regression Model 'Americas SE1'. Related to Results: Spatial Regression Analysis.

Region	Model	LR Test Value	*p*
Americas	Max Age ~ ICM Type	5418.7	<0.001

Table S9. Search terms used in the literature review of Intentional Cranial Modification cases. Related to STAR Methods: Literature Search and Ethical Considerations.

Search term in English
Artificial cranial deformation / Artificial cranial modification
Intentional cranial deformation / Intentional cranial modification
Artificial skull deformation / Artificial skull modification
Intentional skull deformation / Intentional skull modification
Cultural cranial modification
Cranial vault deformation / Cranial vault modification
Cranial deformation / Cranial modification
Deformed skull / Deformed crania / Deformed cranial vault
Modified skull / Modified crania / Modified cranial vault
Tabular deformation / Tabular modification
Tabular erect / Tabular oblique
Oblique deformation / Oblique modification
Annular deformation / Annular modification
Annular erect / Annular oblique
Circular deformation / Circular modification
Circular erect / Circular oblique
Bilobed / Trilobed
Longhead / Elongated
Skull shaping / Skull binding / Skull moulding
Head shaping / Head binding / Head moulding
Archaeological skull
Bioarchaeology / Bioanthropology
Forensic archaeology / Forensic anthropology / Physical anthropology
Skeletal assemblage / Skeletal remains / Human remains
Burial / Burial assemblage / Burial Report
Excavation / Excavation Report
Osteological analysis / Osteological Report
Anthropological analysis / Physical anthropological analysis
Identity / Cultural identity / Social identity
Cultural deformation / Cultural modification / Cultural practice
Eurasia / Europe / Asia / Oceania etc
Hungary, Georgia, France, Australia, Melanesia, Polynesia
Carpathian Basin, Danube Basin
Hun, Ostragoth, Avar
Aztec, Olmec, Taino, Chinook

Table S11. List of localities included in study region. Related to STAR Methods: Construction of the Global Database

Region	Countries
Americas	Argentina, Bahamas, Belize, Bolivia, Canada, Chile, Costa Rica, Cuba, Dominican Republic, Guadeloupe, Guatemala, Jamaica, Lesser Antilles, Mexico, Nicaragua, Peru, Puerto Rico, Suriname, USA, Venezuela, West Indies

Figure S1. Histogram plots showing the distribution of cases across time in the a) annular ICM (n=348) and b) tabular ICM (n=1374) datasets. Related to Results: Descriptive Analysis.

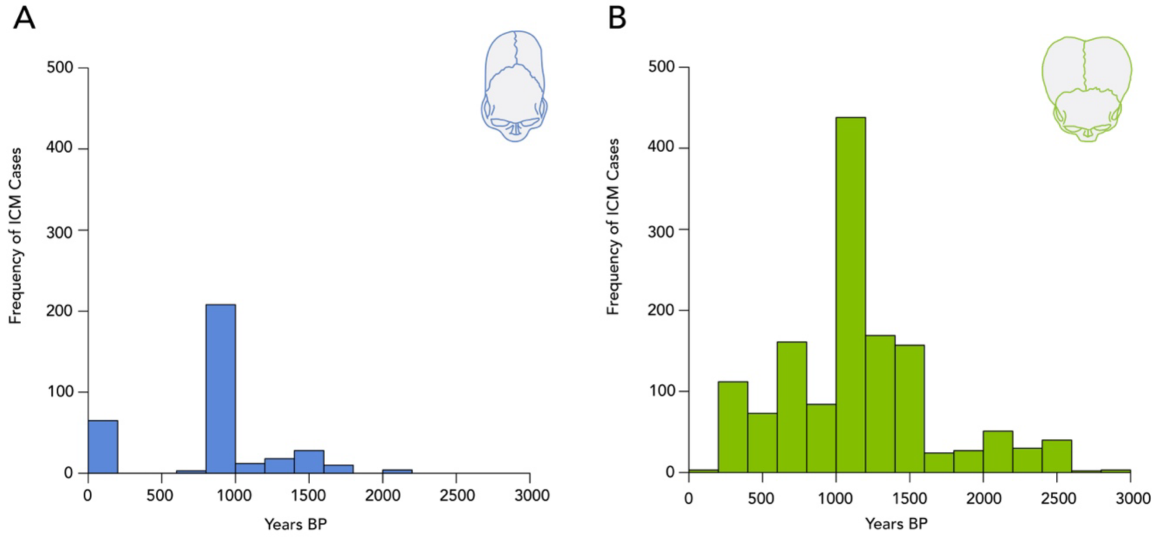

Figure S2. Workflow diagram summarising data preparation and analysis steps undertaken in this study. Related to Results: Descriptive Analysis.

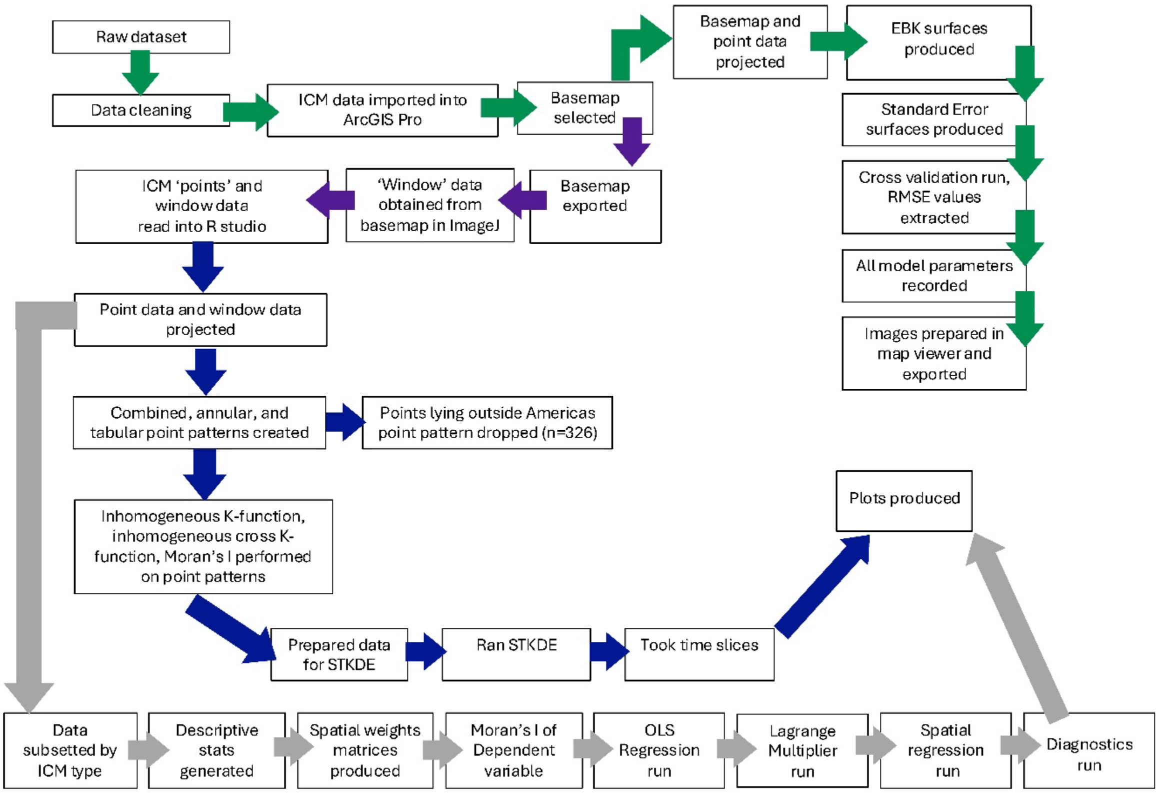

Figure S3. Inhomogeneous *K*-function plots for the Americas a) combined, b) annular, and c) tabular datasets. Related to Results: Assessment of Spatial Autocorrelation and Non-Stationarity.

a) **b)** **c)**

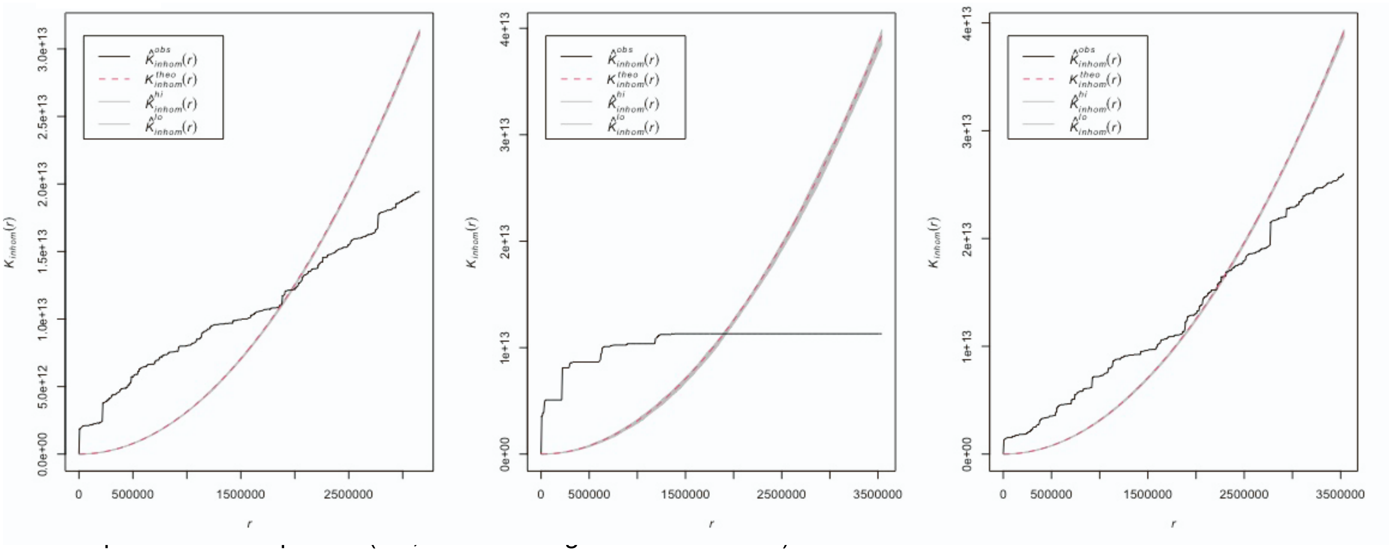

Figure S4. Inhomogeneous cross-*K* function plots for the Americas dataset. Related to Results: Assessment of Spatial Autocorrelation and Non-Stationarity.

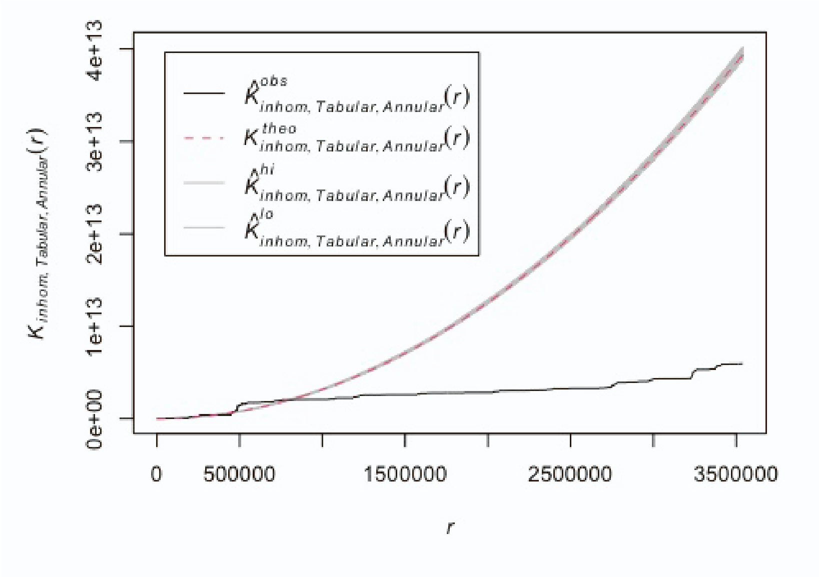

In the Americas, annular cases were significantly more aggregated around tabular cases than expected under CSR until a distance of 500km, after which cases of annular ICM were significantly more dispersed than expected.

Figure S5. Empirical Bayesian Kriging prediction surface for tabular ICM in the Americas. Numbers represent age (BP), with warmer colours (red) denoting older age and cooler colours (blue) denoting more recent ages. Related to Results: Empirical Bayesian Kriging.

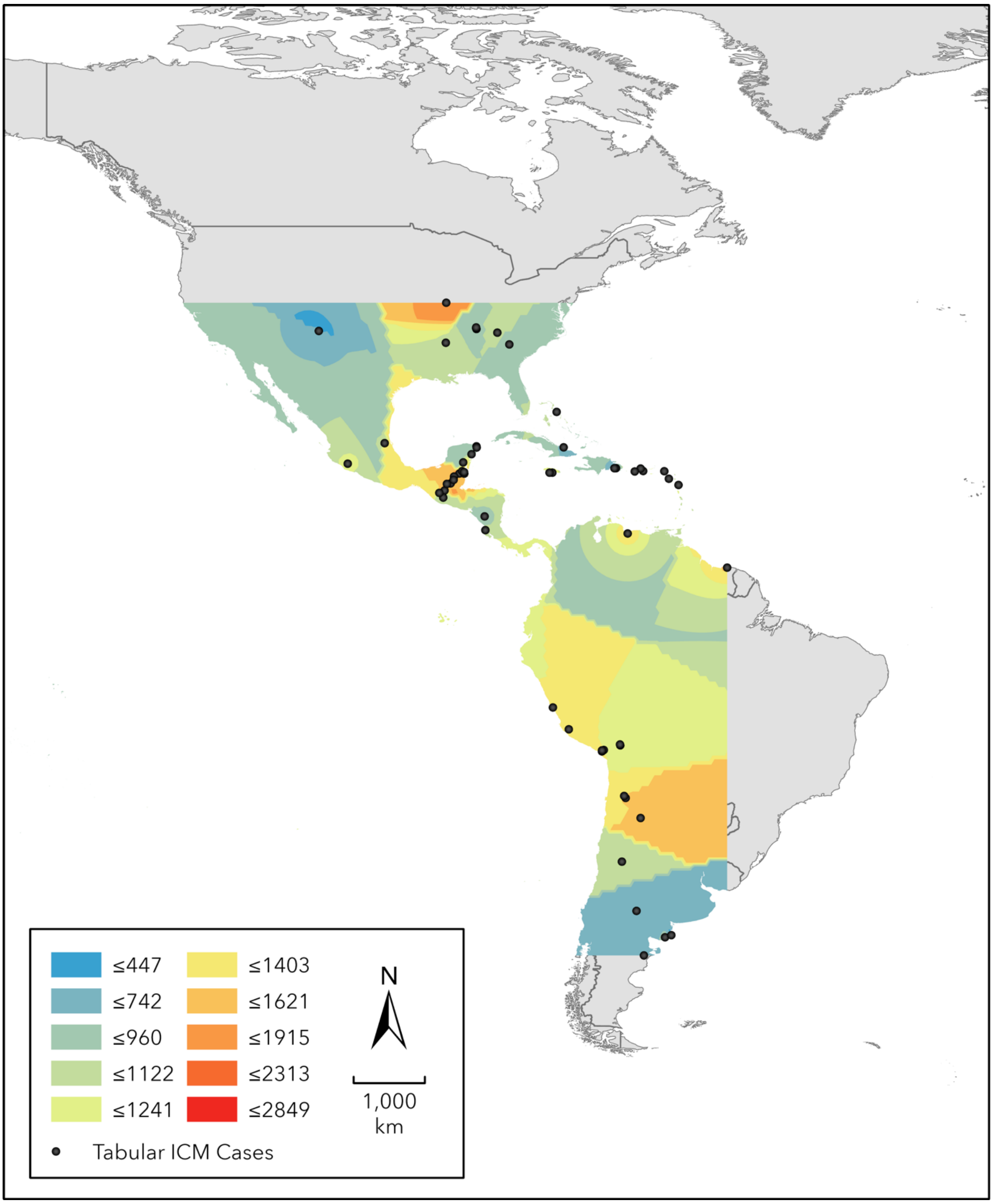

Figure S6. Empirical Bayesian Kriging prediction surface for annular ICM in the Americas. Numbers represent age (BP), with warmer colours (red) denoting older age and cooler colours (blue) denoting more recent ages. Related to Results: Empirical Bayesian Kriging.

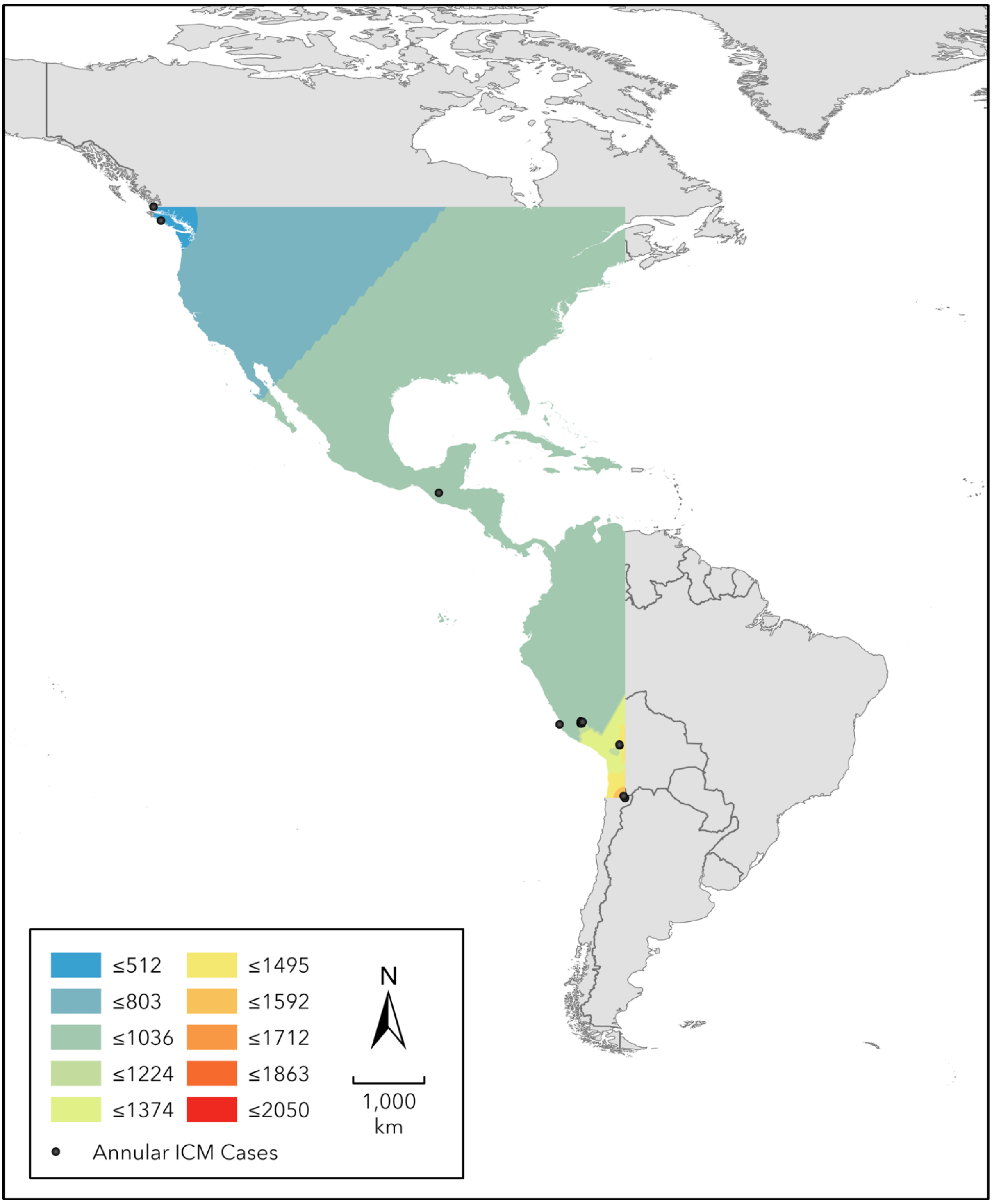

Figure S7. Empirical Bayesian Kriging Standard Error surface for tabular ICM in the Americas. Numbers represent standard error (in years) for the predictions at each location with darker colours denoting higher errors and lighter colours denoting lower errors. Related to Results: Empirical Bayesian Kriging.

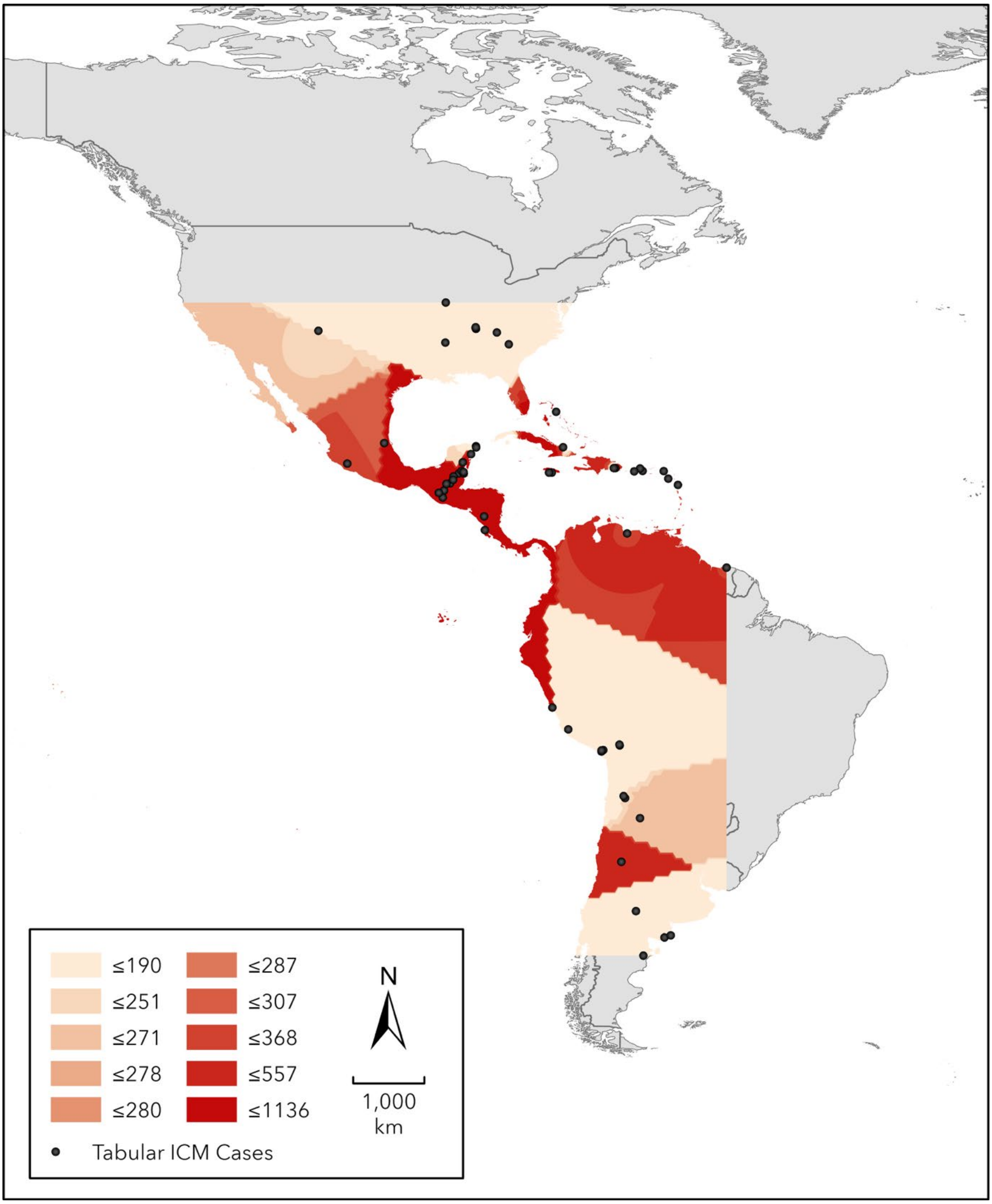

Figure S8. Empirical Bayesian Kriging Standard Error surface for annular ICM in the Americas. Numbers represent standard error (in years) for the predictions at each location with darker colours denoting higher errors and lighter colours denoting lower errors. Related to Results: Empirical Bayesian Kriging.

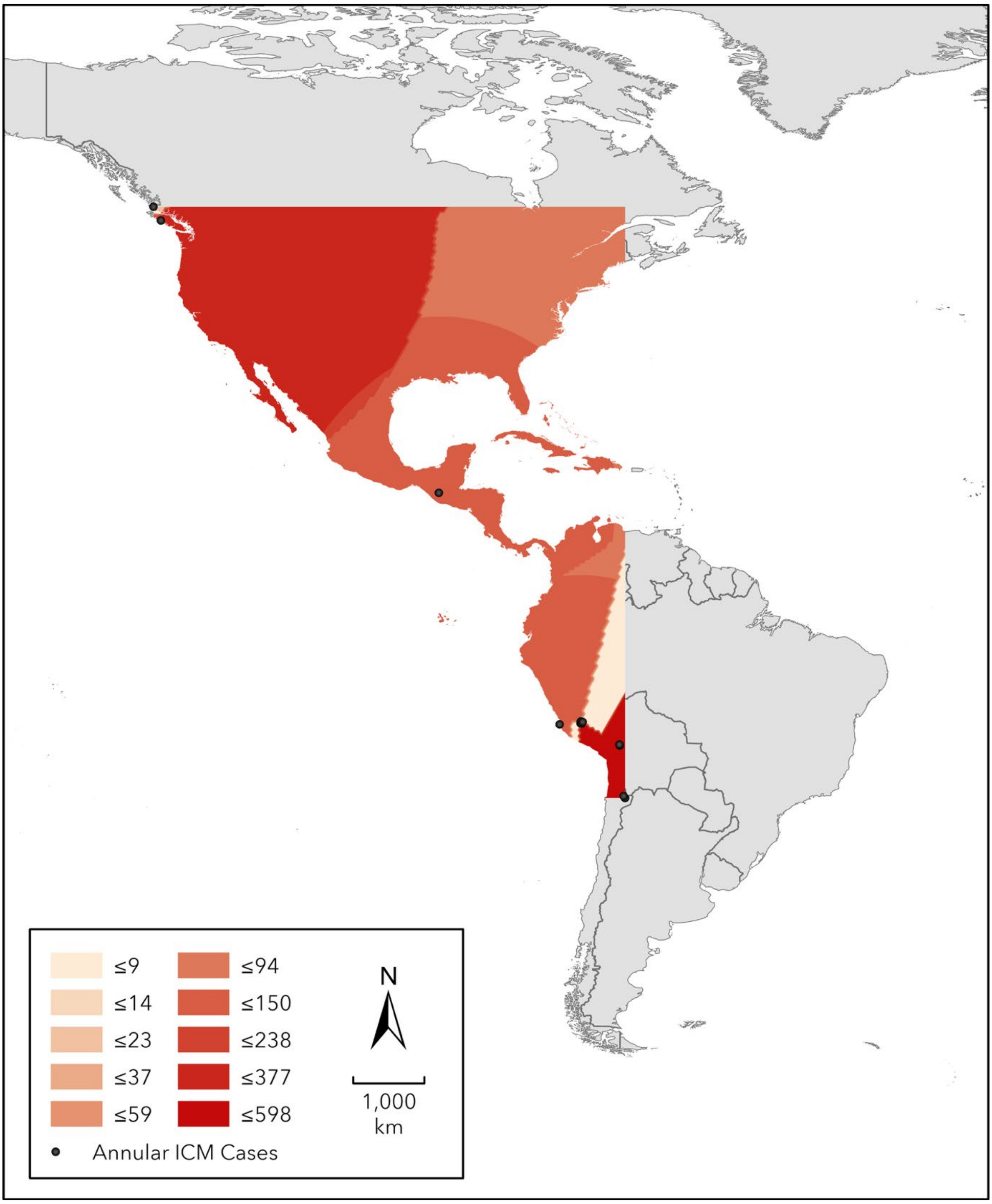

Figure S9. Spatial simultaneous autoregressive error model diagnostics (residual vs fitted value plots, histograms of residuals, and QQ plots for the Americas SE1 model. Related to Results: Spatial Regression Analysis.

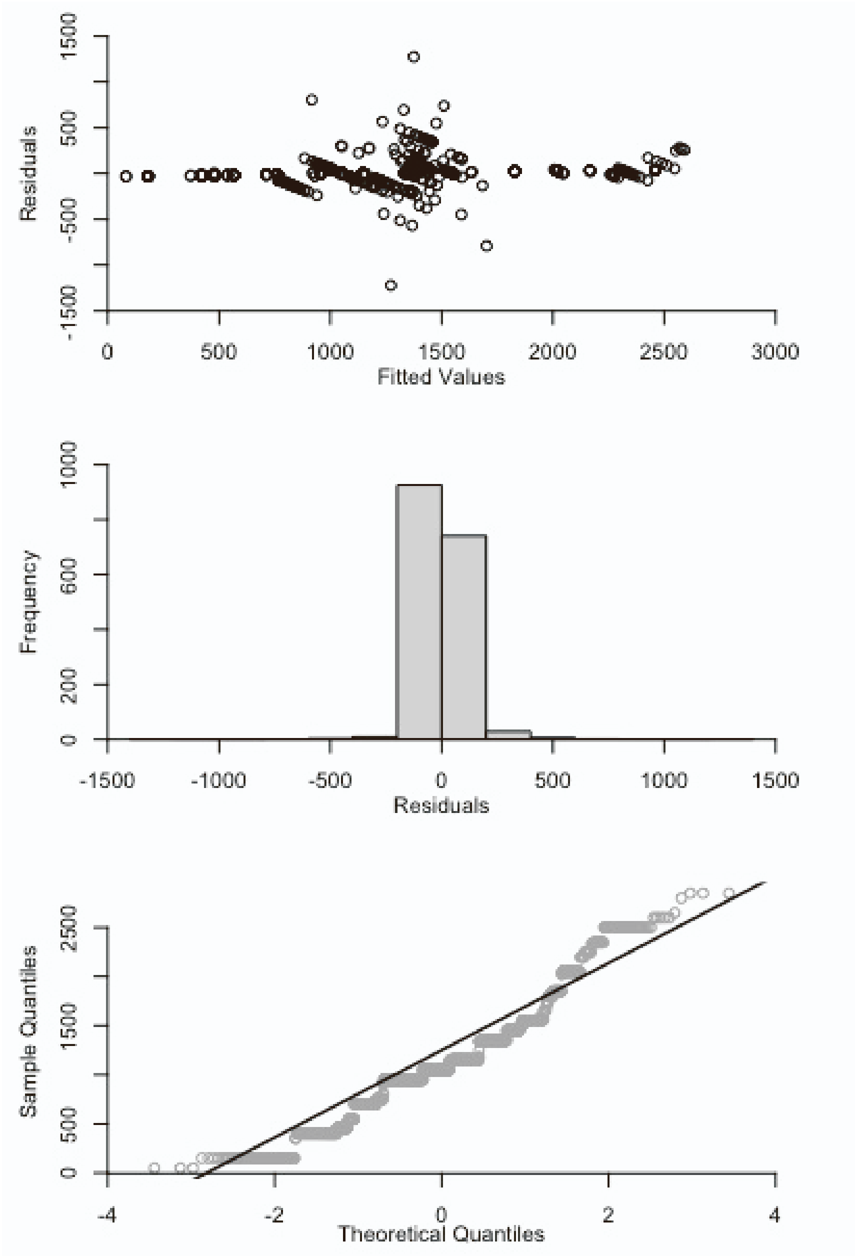

Supplemental References

1. Anselin, L. (2005). Interactive Techniques and Exploratory Spatial Data Analysis. In Geographical Information Systems: Principles, Techniques, Management and Applications, P.A. Longley, M.F. Goodchild, D.A. Maguire, and D.W. Rhind, eds. (John Wiley & Sons), pp. 253-266.
2. Pfeiffer, D.U., Robinson, T.P., Stevenson, M., Stevens, K.B., Rogers, D.J., and Clements, A.C.A. (2008). Spatial Analysis in Epidemiology (Oxford University Press). 10.1093/acprof:oso/9780198509882.001.0001.
3. Agarwal, S.C. (2024). The bioethics of skeletal anatomy collections from India. Nature Communications *15*, 1692. 10.1038/s41467-024-45738-6.
4. Agarwal, S.C. (2025). The disposability and inclusion of Brown bodies. American Journal of Biological Anthropology *186*, e25003. https://doi.org/10.1002/ajpa.25003.
5. de la Cova, C., Hofman, C.A., Marklein, K.E., Sholts, S.B., Watkins, R., Magrogan, P., and Zuckerman, M.K. (2024). Ethical futures in biological anthropology: Research, teaching, community engagement, and curation involving deceased individuals. American Journal of Biological Anthropology *185*, e24980. https://doi.org/10.1002/ajpa.24980.
6. Lambert, P.M., and Walker, P.L. (2018). BIOARCHAEOLOGICAL ETHICS. In Biological Anthropology of the Human Skeleton, pp. 1-42. https://doi.org/10.1002/9781119151647.ch1.
7. Robbins Schug, G., Halcrow, S.E., and de la Cova, C. (2025). They Are People Too: The Ethics of Curation and Use of Human Skeletal Remains for Teaching and Research. American Journal of Biological Anthropology *186*, e70013. https://doi.org/10.1002/ajpa.70013.
8. Zuckerman, M.K., Marklein, K.E., Austin, R.M., and Hofman, C.A. (2025). Exercises in ethically engaged work in biological anthropology. American Journal of Biological Anthropology *186*, e25015. https://doi.org/10.1002/ajpa.25015.
9. Rangel-de Lázaro, G., and Sánchez-Villagra, M.R. (2025). Digitising the past, preserving the future: creating a digital collection of pre-Columbian crania excavated one hundred-twenty-one years ago by Alfredo Jahn in Lake Valencia, Venezuela. STAR: Science & Technology of Archaeological Research *11*, e2488083. 10.1080/20548923.2025.2488083.
10. Alves Cardoso, F. (2026). Data Caring While Caring for Human Remains: Challenges of Legacy Collections. Nature Anthropology *4*, 10001.
11. Gupta, N., Blair, S., and Nicholas, R. (2020). What We See, What We Don’t See: Data Governance, Archaeological Spatial Databases and the Rights of Indigenous Peoples in an Age of Big Data. Journal of Field Archaeology *45*, S39-S50. 10.1080/00934690.2020.1713969.
12. Lien-Talks, A. (2024). How FAIR Is Bioarchaeological Data: With a Particular Emphasis on Making Archaeological Science Data Reusable. Journal of Computer Applications in Archaeology. 10.5334/jcaa.154.
13. Lien-Talks, A. (2026). The Future of Bioarchaeological Data: Why FAIR, CARE, and Machine Learning Are Essential for Sustainable Research. Preprints. Preprints.
14. Gupta, N., Martindale, A., Supernant, K., and Elvidge, M. (2023). The CARE Principles and the Reuse, Sharing, and Curation of Indigenous Data in Canadian Archaeology. Advances in Archaeological Practice *11*, 76-89. 10.1017/aap.2022.33.
15. Carroll, S.R., Garba, I., Figueroa-Rodríguez, O.L., Holbrook, J., Lovett, R., Materechera, S., Parsons, M., Raseroka, K., Rodriguez-Lonebear, D., and Rowe, R. (2023). The CARE principles for indigenous data governance. Open Scholarship Press Curated Volumes: Policy.
16. Plemons, A.M., and Spiros, M.C. (2025). Toward Ethical Digital Practices: Guidelines for Consent, Accountability, and Transparency in Anthropology. American Journal of Biological Anthropology *186*, e70044. https://doi.org/10.1002/ajpa.70044.
